# A Patient-Specific Polylactic Acid Bolus Made by a 3D Printer for Breast Cancer Radiation Therapy

**DOI:** 10.1371/journal.pone.0168063

**Published:** 2016-12-08

**Authors:** So-Yeon Park, Chang Heon Choi, Jong Min Park, MinSoo Chun, Ji Hye Han, Jung-in Kim

**Affiliations:** 1 Department of Radiation Oncology, Seoul National University Hospital, Seoul, Republic of Korea; 2 Institute of Radiation Medicine, Seoul National University Medical Research Center, Seoul, Republic of Korea; 3 Biomedical Research Institute, Seoul National University College of Medicine, Seoul, Republic of Korea; 4 Center for Convergence Research on Robotics, Advance Institutes of Convergence Technology, Suwon, Republic of Korea; Gustave Roussy, FRANCE

## Abstract

**Purpose:**

The aim of this study was to assess the feasibility and advantages of a patient-specific breast bolus made using a 3D printer technique.

**Methods:**

We used the anthropomorphic female phantom with breast attachments, which volumes are 200, 300, 400, 500 and 650 cc. We simulated the treatment for a right breast patient using parallel opposed tangential fields. Treatment plans were used to investigate the effect of unwanted air gaps under bolus on the dose distribution of the whole breast. The commercial Super-Flex bolus and 3D-printed polylactic acid (PLA) bolus were applied to investigate the skin dose of the breast with the MOSFET measurement. Two boluses of 3 and 5 mm thicknesses were selected.

**Results:**

There was a good agreement between the dose distribution for a virtual bolus generated by the TPS and PLA bolus. The difference in dose distribution between the virtual bolus and Super-Flex bolus was significant within the bolus and breast due to unwanted air gaps. The average differences between calculated and measured doses in a 200 and 300 cc with PLA bolus were not significant, which were -0.7% and -0.6% for 3mm, and -1.1% and -1.1% for 5 mm, respectively. With the Super-Flex bolus, however, significant dose differences were observed (-5.1% and -3.2% for 3mm, and -6.3% and -4.2% for 5 mm).

**Conclusion:**

The 3D-printed solid bolus can reduce the uncertainty of the daily setup and help to overcome the dose discrepancy by unwanted air gaps in the breast cancer radiation therapy.

## Introduction

High energy photon beams for radiation therapy exhibit penetration, beam uniformity, and skin-sparing properties [1, 2]. This skin sparing near the surface inside a patient is caused by a dose build-up effect of megavoltage photon beam. The absorbed dose increases within a certain depth beyond the surface until they reach a maximum before megavoltage photon beam reaches electron equilibrium [3]. The ability to spare the skin is very useful for many different types of cancer, however, there is a problem with the treatment of superficial lesions near the skin surface. Thus, a build-up material (bolus) is placed in direct contact with the patient’s skin surface in order to increase the superficial dose and improve dose uniformity by compensating for missing tissue [4–6]. Normal organs including lung and heart could be protected because bolus brings isodose lines toward the surface and it was demonstrated that the use of bolus in the postmastectomy radiotherapy reduced the normal tissue complication probabilities (NTCPs) of the ipsilateral lung [7]. There are several bolus products currently available which are soft and rubbery tissue equivalent materials [8]. In case of the irregular surface region of a patient, unwanted air gaps under bolus might occur between the bolus and patient skin due to the malleability of bolus material.

Bustson *et al*. reported effects on skin dose from air gaps under bolus in the circumstances of breast cancer treatment [9]. That study was performed by different air gaps and oblique incident beams in a flat solid water phantom. Results of the larger air gap showed that the larger reduction of skin dose. The use of a solid bolus placed directly on the patient’s skin has been designed to deliver the desired dose to surface [10]. With manufacturing a solid bolus, several studies have shown the clinical efficacy for head and neck, post mastectomy irradiations, and paraspinal muscle treatment [11–15]. Using 3D printer techniques for radiation therapy has been gaining interest and the 3D printer can create a solid bolus without air gaps ideally.

The aim of this study was to assess the feasibility and advantages of a solid breast bolus made using a 3D printer technique. The bolus substance must be odorless, non-sticky, and harmless to the skin. In this study, thus, the polylactic acid (PLA) was adopted as a bolus material [16, 17]. We printed a patient-specific breast bolus for an anthropomorphic phantom and simulated a real patient treatment. The effects of different breast sizes and bolus thicknesses on the dose distribution were investigated between the commercial bolus and 3D-printed solid bolus.

## Materials and Methods

### Treatment simulation and planning

We used the anthropomorphic phantom (Model 702 Adult ATOM Female; CIRS Inc., Norfolk, VA) with breast attachments for our subject of study. This female phantom with breast attachments of various volumes (200, 300, 400, 500 and 650 cc) underwent the computed tomography (CT) scans with the Brilliance CT Big Bore^TM^ (Philips, Amsterdam, Netherlands). These CT sets with no bolus material (CT_no_) were used to create the virtual bolus structure in the treatment planning system (TPS) and to design the 3D printing bolus. Furthermore, the female phantom, which the commercial bolus (Super-Flex bolus, Action Product Inc., Hagerstown, MD) was placed directly on the breast attachment, underwent other CT scans (CT_bolus_) in order to investigate the effect of unwanted air gaps near the breast surface and nipple. Two boluses of 3 and 5 mm thicknesses were selected for this study.

Conventional radiotherapy treatment of breast cancer used parallel opposed tangential fields as a primary plan to encompass the breast and then one electron beam were used for boost plan [18]. We simulated the treatment for a right breast patient. The primary plans were analyzed using 10 MV photon beams due to large breast volume for this study. The different dynamic wedge filters for different breast attachments were used to increase the dose in the ventral part of the breast while decreasing the dose in the dorsal part. Treatment plans were generated for all breast attachments with different bolus types. The plan__VB_ as an original plan was consisted of CT_no_ sets and virtual bolus structures of water equivalent. The prescription dose to for a whole breast was 50.4 Gy in 28 fractions. Then, the plan__PLA_ was generated in CT_no_ sets with virtual PLA bolus structures. The plan__bolus_ was generated in CT_bolus_ sets including the Super-Flex bolus. The planning parameters of the plan__VB_ were applied to Plan__PLA_ and Plan__bolus_, respectively. Dose distributions for all plans were calculated in Eclipse^TM^ (version A10, Varian Medical Systems, Palo Alto, CA) using the analytical anisotropic algorithm (AAA) with a calculation grid size of 2.5 mm.

### 3D breast bolus design and printing

A patient-specific breast bolus was manufactured using a 3D printer with a clear-PLA material. The design of the breast bolus for 3D printing was based on a CT image. The TPS imported the CT image sets without bolus materials. The boluses of 3 and 5 mm thicknesses were generated as a structure for each CT image set of different breast attachments and the shapes of these boluses were optimized to cover a whole breast without air gaps in the TPS. The bolus material generated in the TPS was set as water-equivalent. We used the clear-PLA material for the plastic material, however, its physical properties were not identical to those of water. The physical properties for the material are listed in Table 1. Thus, the water-equivalent depth of PLA was simply calculated with the ratio of water and PLA physical densities. The bolus thicknesses of 3 and 5 mm for the clear-PLA were reduced to 2.50 and 4.12 mm with 0.83 of the depth-scaling factor, respectively. A Hounsfield unit (HU) of clear-PLA also was set as 274 HU that was derived from an electron density ratio of 1.14 and mass density ratio of 1.2 compared to water.

**Table 1:** Physical properties of the clear-PLA and 3D printer settings.

Print Setting		First Layer Setting		Physical Property of Clear-PLA	
Bed Temperature	65°C	Bed Temperature	65°C	Chemical Formula	C_3_H_4_O_2_
Extruder Temperature	240°C	Extruder Temperature	240°C	Physical Density	1.2 g/cm^3^
Extrusion Width	0.44 mm	Extrusion Width	0.57 mm	Electron Density ratio compared to water	1.14
Layer Height	0.2 mm	Layer Height	0.1 mm	Effective Z	4.22
Layer Speed(Default Printing Speed)	50 mm/s	Layer Speed	15 mm/s		
Fill Density	1 g/cm^3^				
Fill Pattern	Rectilinear				
Fill Angle	45°				
Perimeter Speed	3.33 mm/s				
Perimeter Extrusion Width(Z axis Movement Speed)	0.44 mm				
Nozzle Diameter	0.4 mm				
Filament Diameter	1.75 mm				
Extrusion Multiplier	1				
Z Offset	-1 mm				

*Abbreviations*: PLA, poly lactic acid

All bolus structures of 10 (two boluses for one breast attachment) were exported to 3DSlicer (www.slicer.org), which is the open source software that converts a structure file to the standard tessellation language (STL) file. The converted STL file was interpreted by the 3D printing software Simplify3D^®^ (www.simplify3d.com) and was sent to our 3D printer (MEISTER, 3D BOX Inc., Incheon, Korea). This 3D printer provides the Fused Deposition Modeling (FDM) technology. The optimized print settings for manufacturing the clear-PLA breast bolus are on the Table 1.

### Evaluation of treatment plans

Treatment plans were generated to investigate the effect of unwanted air gaps between bolus and patient skin on the dose distribution of the whole breast. The planned dose difference was calculated for Plan__PLA_ and Plan__bolus_ compared to Plan__VB,_ respectively. The VeriSoft^TM^ 3.1 (PTW, Freiburg, Germany) selected the center of the whole breast in the sagittal plane to calculate the dose difference without suppressing doses.

**Fig 1:**
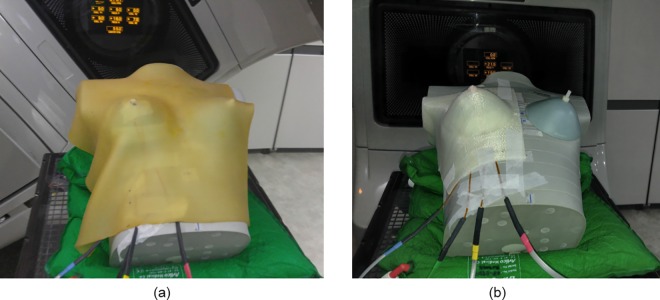
MOSFET placement for measurements under (a) Super-Flex bolus and (b) PLA bolus.

The MOSFET dosimeter (Best Medical Canada, Ottawa, ON, Canada) was used to measure skin doses of breast. The used MOSFET dosimeters were calibrated and the performance characteristics had been verified in the author’s previous study [19]. Both of Plan__VB_ and Plan__PLA_ were applied to measure skin dose, shown in Fig 1. For Plan__VB_, the Super-Flex bolus was used to simulate the treatment condition. The same measurement points located on both sides of the nipple and breast were used for both of Plan__VB_ and Plan__PLA_ with fixed MOSFET positions. Two breast attachments of 200 and 300 cc were used by measuring with different bolus thicknesses of 3 and 5 mm. The Clinac iX (Varian Medical Systems, Palo Alto, CA) delivered the plan. Differences between calculated and measured doses at the surface were acquired to investigate the uncertainty of bolus structures. The percentage difference (%diff) was calculated as follow:

$$\%diff=100\times \frac{Measured\;dose-Calculated\;dose}{Calculated\;dose}$$

## Results

PLA boluses for all breast attachments were successfully manufactured using a 3D printer. Those of 200 and 300 cc volumes were a good fit against the breast of an anthropomorphic phantom without air gaps between the bolus and phantom. For the larger volume, however, we could not lay PLA bolus on the breast because the 3D-printed bolus was a negative mold and the solid bolus did not fit the lower breast where it meets the inframammary fold. Thus, we investigated calculated dose distributions for all breast attachments and both 200 and 300 cc of breast attachments were used to measure skin dose.

### Difference of the dose distribution

**Fig 2:**
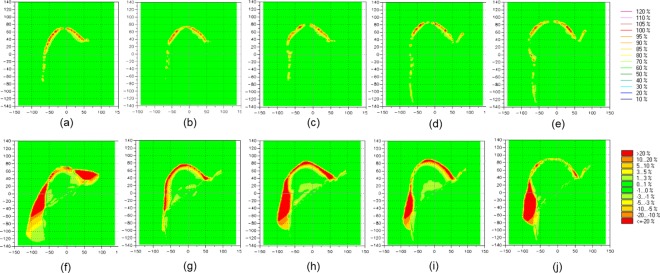
The difference of the dose distribution between Plan__VB_ and Plan__PLA_ for (a) 200 cc,(b) 300 cc,(c) 400 cc, (d) 500 cc, (e) 650 cc attachments, and between Plan__VB_ and Plan__bolus_ for (f) 200 cc, (g) 300 cc, (h) 400 cc, (i) 500 cc, (j) 650 cc attachments using 3-mm-thick bolus.

**Fig 3:**
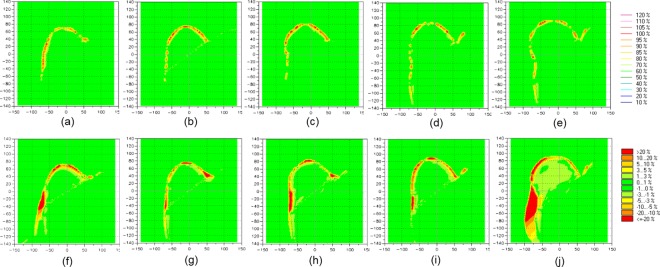
The difference of the dose distribution between Plan__VB_ and Plan__PLA_ for (a) 200 cc,(b) 300 cc,(c) 400 cc, (d) 500 cc, (e) 650 cc attachments, and between Plan__VB_ and Plan__bolus_ for (f) 200 cc, (g) 300 cc, (h) 400 cc, (i) 500 cc, (j) 650 cc attachment using 5-mm-thick bolus.

The planar dose distributions of Plan__VB_ using the virtual water-equivalent bolus were compared to dose planes calculated of treatment plans using PLA bolus and Super-Flex bolus, respectively. Two bolus thicknesses of 3 and 5 mm were then used in treatment planning. Fig 2 shows the comparison of the dose distribution in treatment planning using a 3 mm thick bolus. Compared to Plan__PLA_, the dose difference was shown in the bolus area. The PLA bolus was set to high density material as 274 HU and a thickness was thinner than that of water. Thus, the higher dose was absorbed in the PLA bolus compared to a water-equivalent bolus. The larger volume of the breast shows the greater number of points of the dose difference. It is merely the difference to bolus size to cover the breast. Plan__PLA_ shows no difference in dose to the target of a whole breast. However, compared to Plan__bolus,_ the dose difference due to unwanted air gaps was shown within bolus and breast. The dose difference under the bolus was more than 20% of a normalization value of the prescription for all attachments. The air gap reduced the dose uniformity to the breast and the difference was less than 5% with no impact on the volume size. Fig 3 shows the comparison of the dose distribution in treatment planning using a 5 mm thick bolus. Compared to Plan__PLA_, the dose difference was also shown in the bolus area. There was no differences according to the bolus thickness. However, compared to Plan__bolus,_ the dose difference between bolus and breast decreased compared to 3 mm bolus. A heavier bolus may be described that serve to reduce the air gap through better contact. The numerical data representing percentage of dose difference less than 3% between Plan__PLA_ and Plan__VB_, and Plan__bolus_ and Plan__VB_ for all volumes of attachments are shown in Table 2. The mean percentages of dose difference less than 3% for comparison between Plan__PLA_ and Plan__VB_ (91.5% and 91.4% for 3- and 5-mm-thick bolus, respectively) were higher than those for comparison between Plan__bolus_ and Plan__VB_ (72.9% and 82.0% for 3- and 5-mm-thick bolus, respectively) with statistical significance (*p* < 0.001). There is a good agreement between the dose distribution for the virtual bolus and PLA bolus.

**Table 2:** Percentage of dose difference less than 3% between Plan__PLA_ and Plan__VB_, and Plan__Bolus_ and Plan__VB_ for 200 cc, 300 cc, 400 cc, 500 cc and 650 cc attachments using 3- and 5-mm-thick bolus

	3-mm-thick bolus		5-mm-thick bolus	
	Plan__PLA_ vs. Plan__VB_	Plan__bolus_ vs. Plan__VB_	Plan__PLA_ vs. Plan__VB_	Plan__bolus_ vs. Plan__VB_
200 cc	87.8	65.2	88.9	79.9
300 cc	91.8	72.1	91.4	86.6
400 cc	91.5	72.4	90.4	83.8
500 cc	93.2	76.2	93.0	84.2
650 cc	93.1	78.7	93.5	75.6
Mean	91.5	72.9	91.4	82.0

### Skin dose measurement

**Fig 4:**
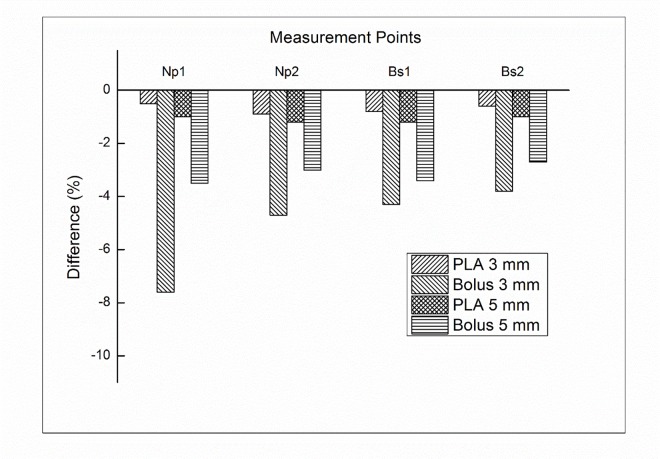
The percentage difference between the calculated and measured doses for Plan__PLA_ and Plan__bolus_ with a 200 cc attachment at both sides of the nipple (Np) and breast surface (Bs).

**Fig 5:**
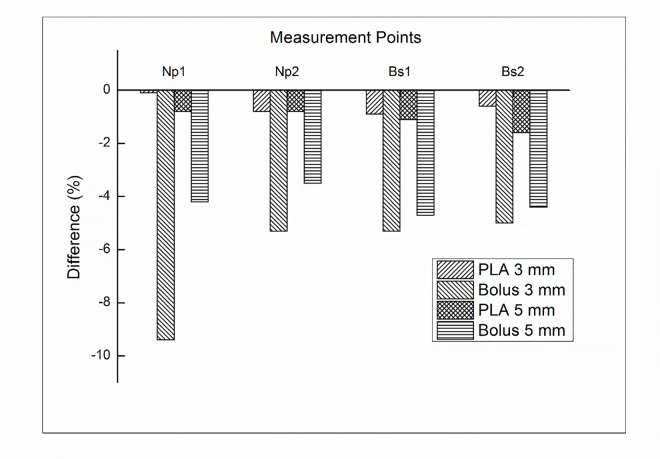
The percentage difference between the calculated and measured doses for Plan__PLA_ and Plan__bolus_ with a 300 cc attachment at both sides of the nipple (Np) and breast surface (Bs).

In addition to dose difference in treatment plan comparison, we further investigated the uncertainty of bolus replacement by measuring the skin dose. Fig 4 shows the percentage difference between calculated and measured doses in 200 cc breast attachment. With PLA bolus of 3 mm, the difference was not significant, which ranged from -0.5% to -0.9%. The average difference in PLA bolus of 5 mm was -1.1%. There was no impact on the volume size. With the Super-Flex bolus, however, significant dose differences were observed. With 3 mm bolus, the average difference was -5.1%, which ranged from -3.8% to -7.6%. With 5 mm bolus, the dose difference was reduced compared to that of 3 mm bolus. The average difference was -3.2%, which ranged from -2.7% to -3.5%. There was still no impact on the volume size. For a 300 cc breast attachment, the average differences were not significant for PLA bolus, which were -0.6% and -1.1% for 3 mm and 5 mm, respectively. With the Super-Flex bolus, the average differences were -6.3% and -4.2% for 3 mm and 5 mm bolus, respectively. The results of the dose difference for a 300 cc breast attachment were shown in Fig 5. The negligible air gap under PLA bolus showed a good agreement between calculated and measured doses regardless of the bolus thickness and breast size. The Super-Flex bolus of 5 mm reduced more air gaps compared to 3 mm bolus due to bolus weight. As a result of the dose difference, the negative values mean that unwanted air gaps make under dose in the measurement.

## Discussion

High energy photon beams have a low dose at the skin surface, which is known as the skin sparing effect. The surface dose predominantly depends on photon beam energy [20, 21]. To cover the tumor which is near the surface, a bolus is used to increase the surface dose. However, commercial flat boluses cannot perfectly contact with the irregular surface, which makes an unwanted air gap between bolus and skin. This air gap significantly affect the surface dose [22]. The bolus routinely is used for radiation therapy of breast cancer. An unwanted air gap might be unavoidable in the daily setup. Moreover, variations in air gaps cannot be predicted in treatment planning. Thus, there is the dose discrepancy between calculated and delivered doses.

This study investigated the feasibility and advantages of a solid breast bolus made using a 3D printer technique. The printing material was the clear-PLA, which had been evaluated in previous other studies as a bolus material [23]. The bolus structure for printing was generated automatically above the body structure following the shape of the patient’s skin in the TPS. The TPS defines the body structure based on the CT image with HU threshold values. A characteristic of all imaging methods is that there is some blurring that occurs within the process. In the CT imaging process, there are several sources of blurring that collectively limit visibility of detail [24, 25]. There was no blurring effect by breathing since the anthropomorphic phantom was used in this study. However, there was still blurred images for the anthropomorphic phantom due to other sources during the imaging process. The blurred boundaries of CT images were included in the body structure. In this study, thus, the appropriate HU threshold value was investigated. The HU value of -950 seems appropriate to print a good fit of PLA bolus.

All of the printed PLA boluses were negative molds and rigid boluses. The breast volumes of bigger than 400 cc are droopy breasts in the anthropomorphic phantom model used in this study. The 3D-printed PLA bolus could not fit the breast attachments bigger than 400 cc because the PLA boluses were not flexible and those breast attachments were also rigid and had breast sagging (ptosis). For the patient with droopy breasts, however, the 3D-printed breast bolus might be applied for radiation treatment in the prone position because human’s breasts feature flexible.

In the case of postmastectomy radiotherapy without reconstruction in breast cancer, commercial bolus is often used to deliver the uniform dose distribution to chest wall [26, 27]. After the mastectomy without breast reconstruction, breast volume is less than 200 cc which may result in small air gap between bolus and patient’s skin. It is different experimental situation from this study. Further study validating the PLA bolus in case of breast volume less than 200 cc will be performed as a future work.

Using 3D printer techniques for radiation therapy have been gaining interest and the clinical efficacy have been investigated in several studies including this study. The 3D printing technique allows to manufacture the patient-specific bolus at low cost. PLA material of one kg used in this study is cheap (less than 30$) and can make two 5-mm-thick bolus. It have taken 12 hours to manufacture one PLA bolus. In order to implement the 3D-printed bolus in a clinic, however, practical problems should be considered such as a clinical process, space, and human resources. Furthermore, the patient may feel discomfort due to the hardness of the plastic bolus which is also limited to sensitive skin or open wounds and 3D-printed PLA bolus may be not well fitted to patient’s skin when patients breathe during treatment. Further work will investigate the soft patient-specific bolus using natural organic polymers such as glucomannan, curdlan, and gelatin to fix these limitation in clinic [8]. At that time, the 3D printer will manufacture the molding box for a patient-specific bolus. In this study, the measurements were performed for only anthropomorphic female phantom. It is important to evaluate the effect of 3D-printed PLA boluses on dose distribution in real cases for daily treatments. Further study evaluating 3D-printed PLA bolus in the case of anatomical modification will be performed with showing statistical analysis.

## Conclusions

The 3D-printed solid bolus was applied to breast radiotherapy. This bolus can reduce the uncertainty of the daily setup and help to overcome the dose discrepancy due to unwanted air gaps. This negative 3D printing mold has a good fit against the irregular breast except for the folded breast. The 3D-printed bolus could replace currently used commercial boluses.

## Supporting Information

S1 Data Dose distributions from Plan_PLA, Plan_VB and Plan_bolus for 200 cc, 300 cc, 400 cc, 500 cc and 650 cc attachments using 3- and 5-mm-thick bolus. (DICOM)
(ZIP)
